# Spatial differentiation of soil nutrients and their ecological chemometrics based on geographic detector in National Agricultural Park of Tangchang, Southwest China

**DOI:** 10.1371/journal.pone.0294568

**Published:** 2024-01-02

**Authors:** Jiufen Liu, Cang Gong, Shunxiang Wang, Liang Wang, Changhai Tan, Lang Wen, Haichuan Lu

**Affiliations:** 1 China University of Geosciences, Beijing, China; 2 National Research Center for Geoanalysis(Key Laboratory of Eco-geochemistry, Ministry of Natural Resources), Beijing, China; 3 Key Laboratory of Natural Resource Coupling Process and Effects, Beijing, China; 4 Natural Resources Comprehensive Survey Command Center of China Geological Survey, Beijing, China; 5 Research Center of Applied Geology of China Geological Survey, Chengdu, Sichuan, China; University of Ferrara, ITALY

## Abstract

In order to analyze the spatial variability of soil nutrients and their ecological chemometrics in Tangchang Town, National Agricultural Park, 20 influencing factors were selected: soil pH, Cd, Hg, As, Cu, Pb, Cr, Zn, Ni, Se, elevation, slope, aspect, land use type, distance from industrial land, distance from commercial land, distance from railway, distance from residential area, distance from highway and distance from river. The effects of various influencing factors on the spatial variability of total organic carbon (TOC), total nitrogen (N), total phosphorus (P), total potassium (K) and ecological stoichiometry were analyzed by means of geographic detector. The results showed that average contents of soil TOC, N, P and K in the study area are 10.24 g kg^-1^, 1.33 g kg^-1^, 1.14 g kg^-1^ and 23.60 g kg^-1^, respectively, and there were significant differences in the spatial distribution of soil nutrients and their eco-stoichiometry in the study area, and TOC, N, P, K, C/N, C/P, C/K, N/P, N/K and P/K has a significant correlation with each other and most correlation coefficients are above 0.5 or below -0.5. Factor detection showed that soil properties, distance from railway and distance from residential area had the most significant explanatory power to the spatial heterogeneity of soil nutrients and eco-stoichiometry. Interaction detection showed that the interaction between soil properties with other factors was the most important factor affecting the spatial differentiation of soil nutrients and their ecological chemometrics, and elevation, distance from railway and distance from residential area were also important factors. Risk detection showed that the differences of soil nutrients and their ecological stoichiometry were most significant in the subregions of soil properties (pH, Cd, Hg, As, Cu, Pb, Cr, Zn, Ni and Se).

## Introduction

Soil nutrients such as total organic carbon (TOC), nitrogen (N), phosphorus (P) and potassium (K) are the main macronutrients in terrestrial ecosystem, which are closely related to soil fertility, plant growth, environmental problems and biogeochemical cycle [1–3]. Soil nutrient is an important index of land quality evaluation. Due to the impacts of various natural and human factors (such as fertilization, land use change, rain water erosion and soil microbial activities, etc.), soil nutrients show obvious spatial heterogeneity [4–7], which causes uncertainty to the results of land quality evaluation. Therefore, a better understanding of soil TOC N, P, K levels and their spatial variability and their influencing factors is necessary to evaluate soil productivity, guide nutrient management, assess potential environmental pollution and understand biogeochemical cycle. The stoichiometric relationship of soil carbon, nitrogen, phosphorus and potassium is a good indicator, which is of great significance for revealing the mineralization and biogeochemical cycle of carbon, nitrogen and phosphorus, for example, the ratio of soil carbon to nitrogen (C/N) is a sensitive index of soil quality and can reflect the degree of decomposition of organic matter, and C/P is generally regarded as a symbol of soil organic phosphorus mineralization ability [8, 9]. Therefore, the study on the spatial variability and influencing factors of soil nutrients and their ecological chemometrics has important theoretical and practical significance.

In the past few decades, the use of various chemical fertilizers to prevent the lack of soil nutrients, greatly changed the spatial variability of soil nutrients from the field level to the national scale, in addition, the application of soil improver can increase soil nutrients and improve soil quality by improving soil physical, chemical and biological characteristics, but also changed the relationship between soil nutrients [10–12]. Therefore, in recent years, more and more attention has been paid to the analysis of spatial variability of soil nutrients and its influencing factors. For example, Yang [13] and Liu [14] studied the effects of topographic factors such as elevation, slope and aspect on soil nutrient variation, and found that there were different correlations between topographic factors and soil nutrients under different micro-topographic conditions. there are also some differences in the explanatory power of topographic factors to soil nutrient variation. Due to the complexity of terrestrial ecosystem, coupled with the differences of many environmental factors, such as climate, parent material, topography, soil types and human activities, the spatial variability of soil nutrients is different in different regions, and the effects of these environmental factors on the spatial variability of soil nutrients vary with different scales and regions [15–17]. Although many studies have focused on the correlation between soil nutrients and their influencing factors, they are mainly based on the influence degree of single factor, and there is a lack of research on the spatial difference of nutrients by combinatorial variables. On the other hand, most of the studies on soil C, N, P and K nutrient elements focus on the variation characteristics of soil C, N, P and K elements [1–3, 5, 8, 12], while ignoring the coupling equilibrium relationship between soil C, N, P and K. Under the combined action of structural factors and random factors, vegetation, climate, parent material, soil type, topography, biological activities, planting system and fertilizer application rate will have a certain impact on soil C, N, P and K content and its metrological characteristics. Geographical detector [18] as the detection of different scale variables space differentiation characteristics or exploratory spatial data analysis of the consistency between powerful tool, more intuitive, fast and effective measure of the contribution of each factor, there is no strong model assumption, solve the limitations of traditional methods in the analysis category variables [19, 20]. Such as Liu [21] used geographical detectors to study the influencing factors of soil phosphorus loss, and obtained the strong interpretation of single factor and interaction. In addition, it has been widely used in many fields such as underground [22], land use [23], heavy metals [24] and ecological vulnerability [25].

Tangchang Town, the National Agricultural Park, located in the heart of Chengdu Plain, is one of the dominant areas of hotbed chives in the country, also known as the "Hometown of Hotbed Chives ". However, in recent years, with the aggravation of the contradiction between human and land, the improvement of land reclamation coefficient and the unreasonable planting and fertilization methods, the utilization rate of soil chemical fertilizer in this region is not high. Soil consolidation, nutrient imbalance, soil acidification and non-point source pollution are prominent [24, 26, 27]. It is very important to design effective management practices for agricultural activities and environmental protection to evaluate the spatial variability of soil nutrients and their ecological chemometrics by combining soil types, topography and land use types, and soil physical and chemical properties.

Therefore, this study takes Tangchang Town, a national agricultural park in China, as the research object, and makes an in-depth study on the spatial variability and influencing factors of soil main nutrients TOC, N, P, K and their eco-chemometrics by using geographic detectors. The specific objectives are: (1) to understand the spatial distribution characteristics of soil TOC, N, P, K content and their ecological chemometrics; and (2) to determine the dominant factors affecting soil TOC, N, P, K content and their ecological chemometrics spatial variation and their interaction. The results of our study will provide targeted suggestions for agricultural activities and environmental monitoring in the study area.

## Material and methods

### Study area

**Fig 1:**
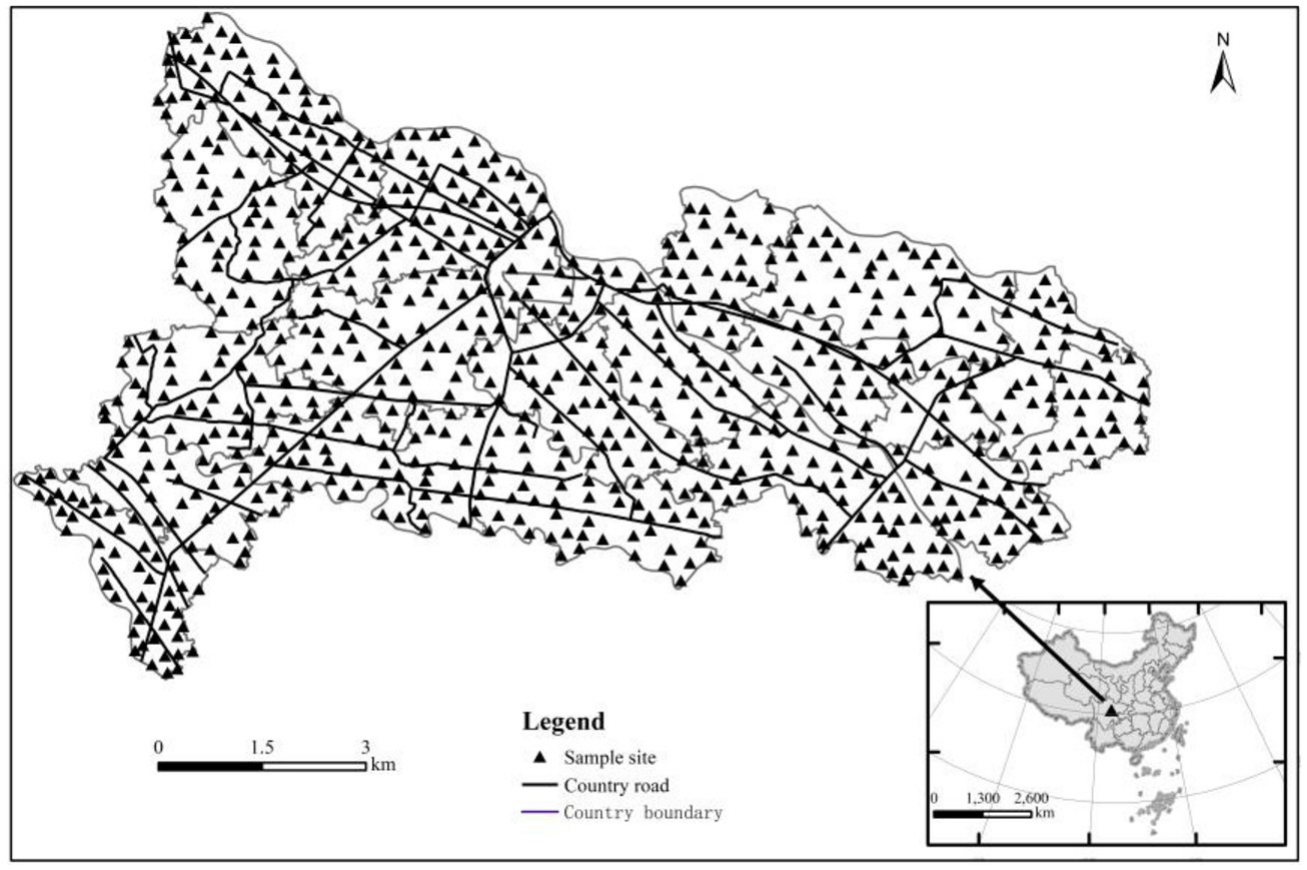
Locations of study area and sampling sites.

As shown in Fig 1, TangChang town is located in the hinterland of Chengdu Plain in southwest China, 30°52’22"-30°56’20" N, 103°44’44"-103°54’51" E. It covers an area of 74.6 km^2^, of which 95% is cultivated land. It has a subtropical monsoon humid climate with an average annual temperature of 15.7°C, an average annual rainfall of 972 mm. Except for a small number of shallow hilly platforms, most of the areas are flat dams, and 90% of the soil is black oil sandy soil formed by impact. 75% of the town is located in the water source protection area, which is the core area of drinking water source protection in Chengdu. Baitiao River, Xuyan River and other major rivers flow through the town area. Major transportation hubs such as Chengguan Expressway, National Highway 317 and Chengguan Express Railway run through the whole territory. The main types of land use are cultivated land (about 46.5%) and woodland (24.1%), followed by residential land (13.9%). 1.9% of the industrial land is scattered in the southwest and central regions. Tangchang Town is one of the top ten agricultural demonstration towns in China, located in the quintessence area of self-flow irrigation in Dujiangyan, an important vegetable basket base in Chengdu and the largest leek production base in southwest China. The "Tangyuan Hotbed Chives Industrial Park" in the town is named as Chengdu four-star modern agricultural demonstration park.

### Sample collection and measurement

Field sampling was performed in April 2021. The sample collection was done based on specification of land quality geochemical assessment (DZ/T 0295–2016), a total of 788 topsoil (0–20 cm) samples were collected. The sampling locations are shown in Fig 1. For detailed sample collection and sample analysis and testing, see our previous research [24, 27, 28].

### Research methods

#### Geographical detector

Geographic detector measure the contribution of independent variables to dependent variables by calculating the ratio of the sum of variances of their respective variables to the sum of variances of dependent variables, including factor detector, interactive detector, risk zone detector and ecological detector [18].

The factor detector is used to detect the spatial differentiation of dependent variables and the ability to explain the influence of respective variables on dependent variables, which is measured by *q* value [18].

$$q=1-\frac{\sum_{h=1}^{L}N_{h}\sigma_{h}^{2}}{N\sigma^{2}}=1-\frac{SSW}{SST}$$ (1)

where h = 1…, L is the classification number of independent variable X, N_*h*_ and N are the number of units in the layer and the whole area, respectively; σ_h_^2^ and σ^2^ are the variances of the classification h and the factor variable Y within the area, respectively. SSW and SST are the sum of all the variances of the independent variable X and the total variance within the area, respectively. The range of *q* is [0,1], the greater the value of *q*, the greater the influence of the independent variable X on the dependent variable Y.

The interaction detector judges the influence degree of the interaction between the independent variables on the dependent variables by identifying the *q* value of the interaction between two different independent variables. Basis for judgment: *q*(*X*_*a*_∩*X*_*b*_) < min[*q*(*X*_*a*_), *q*(*X*_*b*_)] interaction is nonlinear weakening; min min[*q*(*X*_*a*_), *q*(*X*_*b*_)]< *q*(*X*_*a*_∩*X*_*b*_)<max[*q*(*X*_*a*_), *q*(*X*_*b*_)] is one-way nonlinear weakening; *q*(*X*_*a*_∩*X*_*b*_)>max[*q*(*X*_*a*_), *q*(*X*_*b*_)] is two-factor enhancement, *q*(*X*_*a*_∩*X*_*b*_) = *q*(*X*_*a*_)+*q*(*X*_*b*_) is independent interaction, and *q*(*X*_*a*_∩*X*_*b*_)>*q*(*X*_*a*_)+*q*(*X*_*b*_) is nonlinear enhancement.

The risk zone detector is used to detect whether there are significant differences in soil nutrients and eco-chemometrics among the sub-regions of the impact factors, which are tested by *t* statistics.

$$t_{{\bar{y}}_{h=1}-{\bar{y}}_{h=2}}=\frac{{\bar{Y}}_{h=1}-{\bar{Y}}_{h=2}}{{\left[\frac{Var({\bar{Y}}_{h=1})}{n_{h=1}}+\frac{Var({\bar{Y}}_{h=2})}{n_{h=2}}\right]}^{1/2}}$$ (2)

where Y_*h*_ is the attribute mean value in the sub-region *h*, this study is the soil nutrient content and its ecological chemometrics; Var is the variance; n_*h*_ is the sample number in the sub-region *h*; the statistic *t* approximately obeys the Student’s *t* distribution, the higher the *t* value, the greater the influence factor on the spatial diversity of soil nutrients and its eco-chemometrics.

Eco-detector is used to compare whether there are significant differences in the effects of two influencing factors on the spatial distribution of soil nutrients and their eco-stoichiometry, which is measured by *F* statistic:

$$F=\frac{{SSW}_{X_{a}}N_{X_{a}}\left(N_{X_{b}}-1\right)}{{SSW}_{X_{b}}N_{X_{b}}\left(N_{X_{a}}-1\right)}$$ (3)

$${SSW}_{X_{a}}=\sum_{h=1}^{L_{a}}N_{h}\sigma_{h}^{2},{SSW}_{X_{b}}=\sum_{h=1}^{L_{b}}N_{h}\sigma_{h}^{2}$$ (4)

where $N_{X_{a}}$ and $N_{X_{b}}$ are the sample sizes of two independent variables *X*_*a*_ and *X*_*b*_, respectively; ${SSW}_{X_{a}}$ and ${SSW}_{X_{b}}$ represent the sum of intra-layer variances of the layers formed by *X*_*a*_ and *X*_*b*_, respectively; *L*_*a*_ and *L*_*b*_ are that hierarchical number of variables *X*_*a*_ and *X*_*b*_, respectively. Where zero assumes ${H_{0}:SSW}_{X_{a}}={SSW}_{X_{b}}$. If *H*_*0*_ is rejected at the significance level of *α*, it indicates that there are significant differences in the influence of two independent variables *X*_*a*_ and *X*_*b*_ on the spatial distribution of attribute dependent variable *Y*.

#### Factor index selection and data processing

Referring to the selection methods of factors and indicators in the literature [21, 24], combining with the actual situation of the study area, 20 parameters such as soil properties (pH and the contents of Cd, Hg, As, Cu, Pb, Cr, Zn, Ni and Se), topographic factors (elevation(X_1_), slope(X_2_) and aspect(X_3_)), soil-forming factors (land use types(X_4_)) and distance factors (distance from industrial land(X_5_), commercial land(X_6_), railways(X_7_), residential areas(X_8_), roads(X_9_) and rivers(X_10_)) were selected as the factors of this study. When using geographic detectors to analyze influencing factors, the dependent variable must be numerical quantity and the independent variable must be type quantity. If the independent variable is numerical quantity, it needs to be discretized into type quantity [18]. The natural breakpoint method is used to divide 20 influencing factors into 6 categories. SPSS26.0 is used for descriptive statistical analysis and correlation analysis of data, ArcGIS10.8 is used for drawing sampling and spatial distribution, Origin2019 is used for drawing, and GeoDetector software (http://www.geodetector.org/) is used for geographic detector.

## Results

### Statistical characteristics of soil nutrient content and ecological chemometrics

The change and development of soil nutrients is caused by the interaction of biological factors that play a leading role in the process of parent material soil formation, geological large cycle and biological small cycle, the C, N, P and K are the most important biogenic elements of biological organisms, which play an important role in the structure and function of the ecosystem [29]. The statistical results (Table 1) show that the average contents of soil TOC, N, P and K in the study area are 10.24 g kg^-1^, 1.33 g kg^-1^, 1.14 g kg^-1^ and 23.60 g kg^-1^, respectively. According to the classification standard of soil nutrient index (Table 2) [30], the average content of TOC is in the fourth level of the classification standard, which belongs to the relatively lack level; the average content of N is in the third level, belonging to the suitable level; the average content of P is in the first level, belonging to the rich level; the average content of K is in the second level, belonging to the relatively rich level.

**Table 1:** Descriptive statistics of soil C, N, P and K and eco-stoichiometric ratio in study region.

Components	Max	Min	Median	Mean	S.D	CV(%)
P (g kg^-1^)	3.29	0.22	1.11	1.14	0.38	33.37
K (g kg^-1^)	27.98	14.69	24.32	23.60	2.37	10.04
N (g kg^-1^)	2.40	0.70	1.30	1.33	0.26	19.69
TOC (g kg^-1^)	25.41	1.39	10.15	10.24	2.94	28.72
C/N	21.17	1.27	7.72	7.88	2.49	31.63
C/P	38.57	2.17	9.06	9.61	3.39	35.25
C/K	1.06	0.050	0.42	0.44	0.14	31.65
N/P	6.79	0.33	1.20	1.32	0.59	44.90
N/K	0.15	0.028	0.056	0.057	0.013	23.13
P/K	0.14	0.013	0.047	0.048	0.016	33.31

**Table 2:** The classification standard of soil nutrient index.

Parameter	First class (Rich)	Second class (Relatively rich)	Third class (Suitable)	Fourth class (Relatively lack)	Fifth class (Lack)
N	>2	>1.5–2	>1–1.5	>0.75–1	≤0.75
P	>1	>0.8–1	>0.6–0.8	>0.4–0.6	≤0.4
K	>25	>20–25	>15–20	>10–15	≤10
TOC	>40	>30–40	>20–30	>10–20	≤10

Soil C:N:P:K is mainly controlled by hydrothermal conditions, soil formation and soil nutrient characteristics, but due to the influence of soil forming factors (climate, geomorphology, vegetation, parent rock, soil animals, etc.) and human activities, the spatial variation of soil C:N:P:K is large. Different climate, land use intensity, vegetation litter decomposition and biological succession process will change the content and proportion of soil C, N, P, K [29]. The average values of soil C/N, C/P, C/K, N/P, N/K and P/K are 7.88, 9.61, 0.44, 1.32, 0.057 and 0.048 respectively. The variation coefficient of soil C, N, P, K content and its ecological chemometrics in the study area was 10.04%-44.90%, which belonged to the moderate degree of variability.

### Spatial distribution characteristics of soil nutrient content and ecological chemometrics

**Fig 2:**
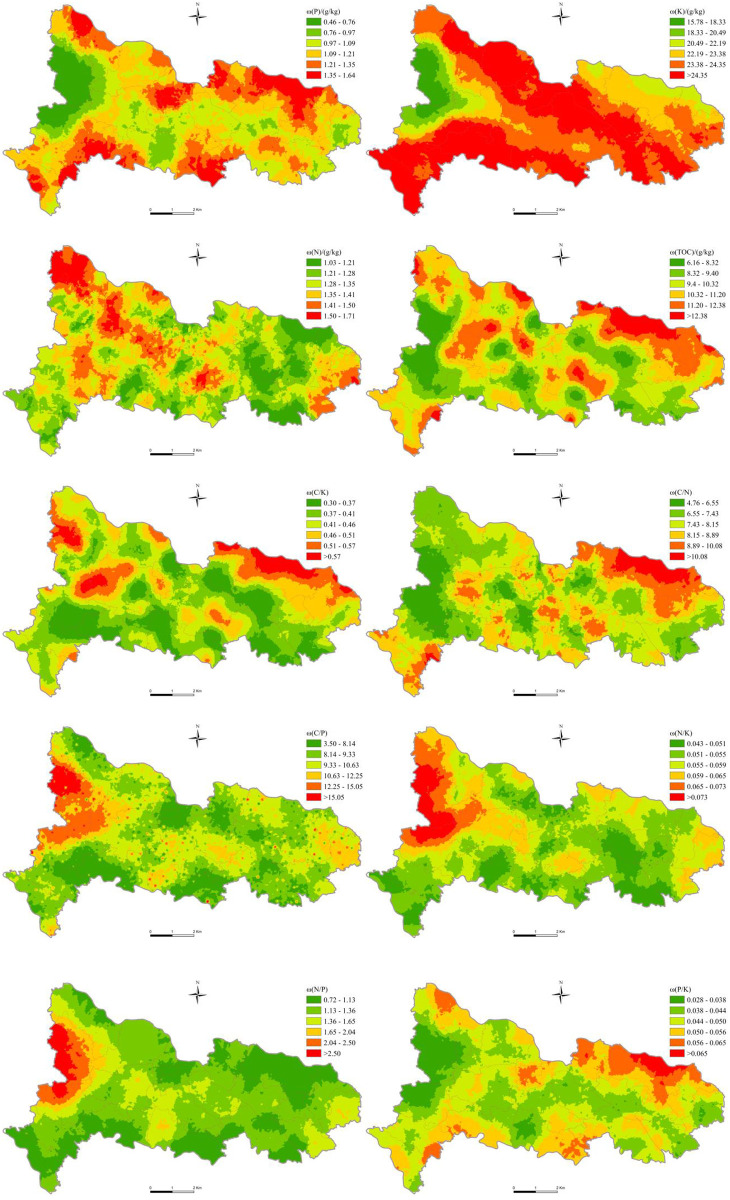
Spatial distribution of the soil nutrients and ecological chemometrics in the topsoil.

Fig 2 shows the spatial distribution of TOC, N, P, K, C/N, C/P, C/K, N/P, N/K and P/K in the topsoil of the study area. It can be seen that the high value area of P is distributed in the northern and southern marginal areas, and the low value area is mainly concentrated in the western villages of the study area; the high value area of K is distributed in most areas except a few villages in the west and northeast; the high value area of N is mainly concentrated in a few areas in the northwest of the study area, while the high value area of TOC is concentrated in the central and northeastern areas. The C/P, N/K and N/P showed similar spatial distribution characteristics, and the higher C/P, N/K and N/P were mainly concentrated in the western part of the study area; the higher C/N and P/K were mainly distributed in the northeast region; the high value area of C/K is mainly distributed in the northeast, northwest and western parts.

### Correlation analysis

**Fig 3:**
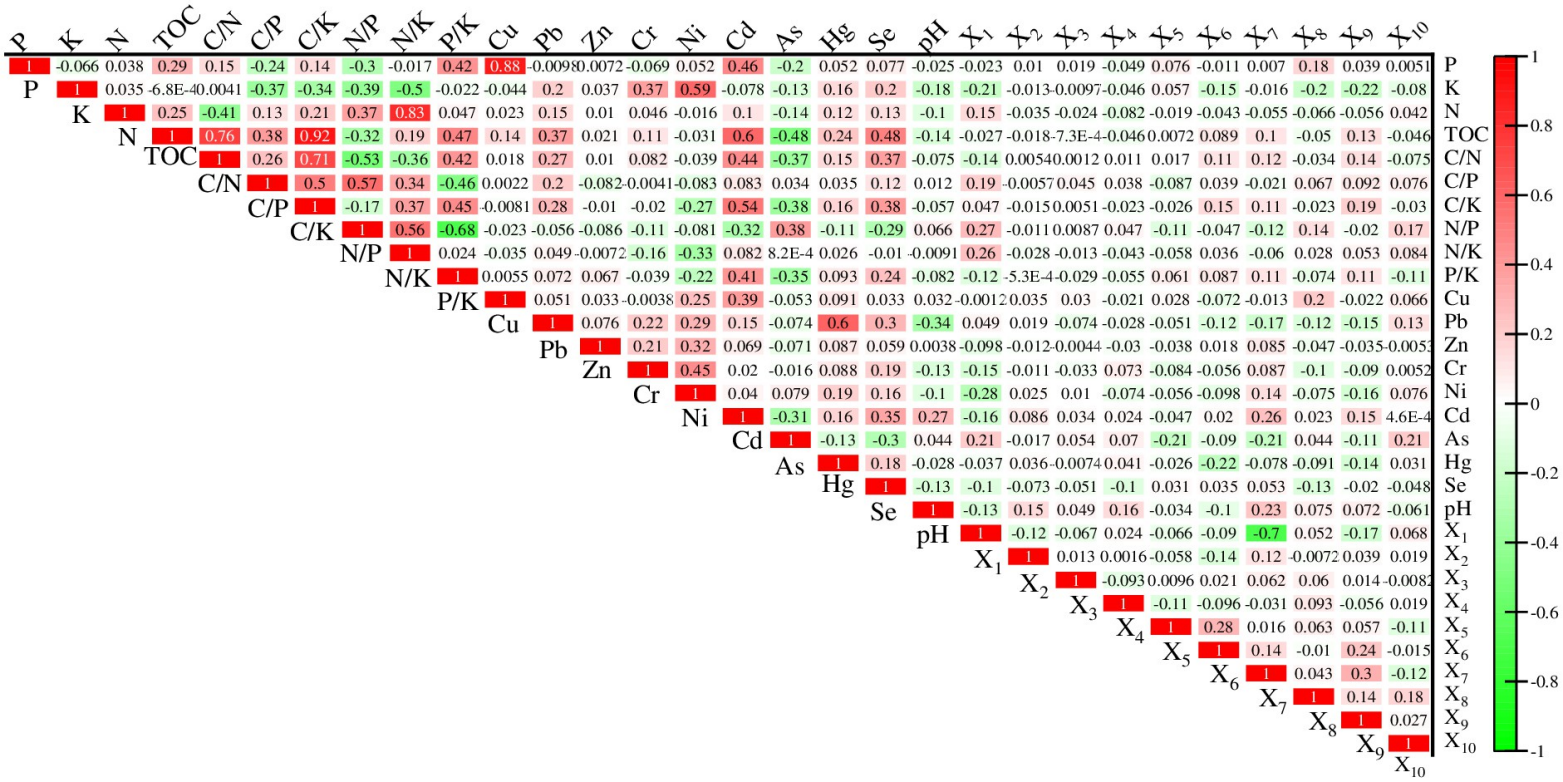
Pearson correlation coefficient of soil nutrients and ecological chemometrics with impact factors.

The correlation analysis results show that most TOC, N, P, K, C/N, C/P, C/K, N/P, N/K and P/K have extremely significant (*p*<0.01) correlations (Fig 3), especially the correlations between TOC-C/N, TOC-C/K, C/N-C/K, C/P-C/K, C/N-N/P, C/P-N/P, N-N/K, N/P-N/K and P/K-N/P are above 0.5 or below -0.5. In soil properties, TOC, N, P, K, C/N, C/P, C/K, N/P, N/K with four or more factors of Cu, Pb, Zn, Cr, Ni, Cd, As, Hg, Se and pH have extremely significant or significant correlation, which indicates that the change of soil properties in the study area is closely related to soil nutrients. Among topographic factors, there is a very significant positive correlation between X_1_ with N, C/P, N/P and N/K, a very significant negative correlation between X_1_ with K, C/N and P/K, but no correlation between X_2_ and X_3_ with soil nutrients and ecological chemometrics. There is no correlation between soil nutrients and ecological chemometrics with X_4_ except N, indicating that soil use types have a weak impact on soil nutrients and ecological chemometrics in the study area. In the distance factor, P has a very significant positive correlation with the X_8_; K has a very significant negatively correlated with the X_6_, X_8_ and X_9_; TOC, C/N and C/K are significantly positively correlated with the X_6_, X_7_ and X_9_; N/P has a significant negative correlation with the X_5_ and X_7_, a significant negative correlation with X_8_ and X_10_; P/K has a very significant negative correlation with the X_10_, and a very significant positive correlation with the X_7_ and X_9_. It can be seen that the distance factor has certain influence on soil nutrients and ecological chemometrics.

### Geoprobe analysis of factors affecting soil nutrients and their ecological stoichiometry

#### Factor detector

Factor detector was used to detect the explanatory power of various influencing factors to soil TOC, N, P, K, C/N, C/P, C/K, N/P, N/K and P/K, and the *q* value was used to measure the explanatory power.

**Fig 4:**
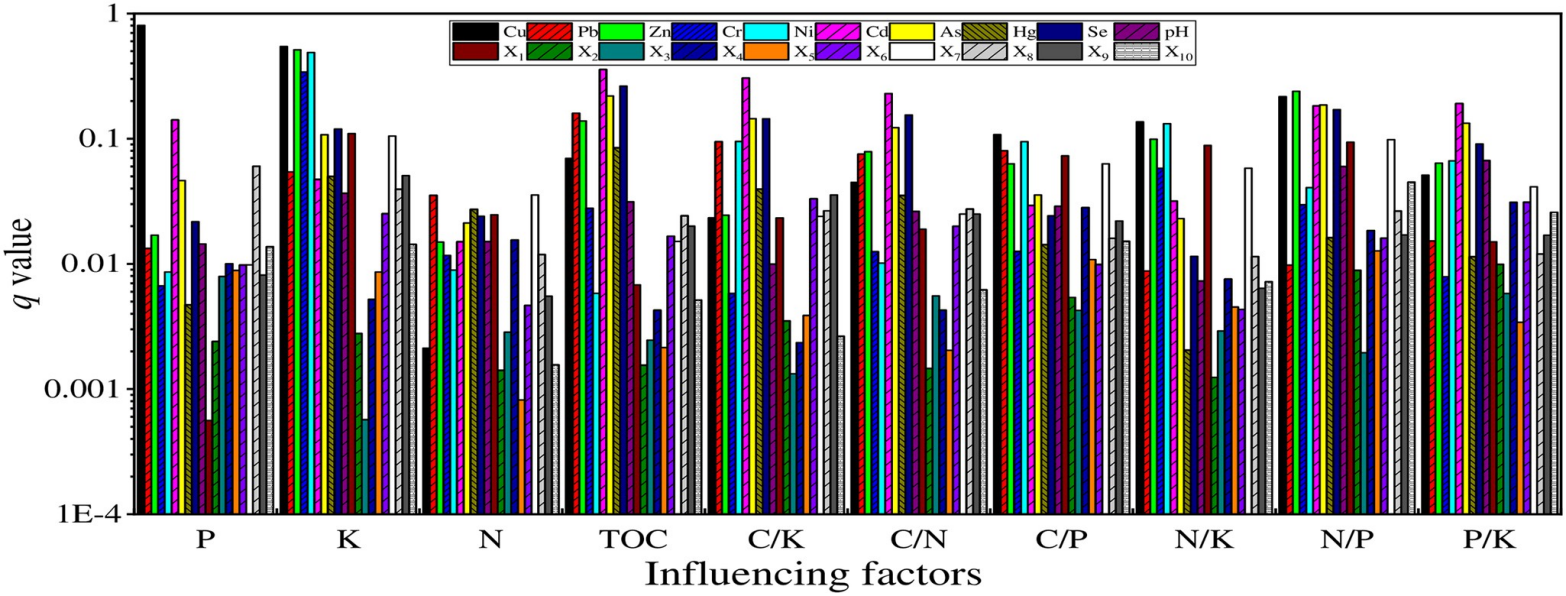
Effects of different factors on the explanatory power of soil nutrients and ecological chemometrics with *q* value.

The operation results of factor detector showed that there were differences in the explanatory power of 20 influencing factors to soil nutrients and eco-stoichiometry (Fig 4): the primary influencing factor of P was soil Cu content (0.804), followed by Cd content (0.141), followed by X_8_ (0.060); the three main factors of K spatial variation were soil Cu content (0.545), Zn content (0.512) and Ni content (0.488); the first influencing factor of N is the X_7_ (0.036), the second is Pb content (0.035), and the third is the influence of Hg content (0.027); the three main factors causing the spatial variation of TOC, C/K, C/N and P/K are soil content of Cd, Se and As; the first influencing factor of C/P is Cu content (0.108), the second is Ni content (0.095), and then Pb content (0.080); the three main factors causing the spatial variation of N/K are content of Cu(0.136), Ni(0.132) and Zn(0.099); the three main factors causing the spatial variation of N/P are content of Zn(0.239), Cu(0.217) and As(0.186).

#### Interaction detector

The composition and structure of soil are complex. The spatial distribution of soil C, N, P, K and their ecological chemometrics is usually formed by the joint action of multiple factors. It is impossible for a single factor to affect the distribution and change of soil nutrients and their ecological chemometrics. Therefore, using the interaction detector to analyze the interaction degree of various factors on the spatial distribution of soil C, N, P and K and their ecological chemometrics is conducive to accurately determine the deep driving mechanism affecting the spatial distribution of soil C, N, P and K and their ecological chemometrics.

**Fig 5:**
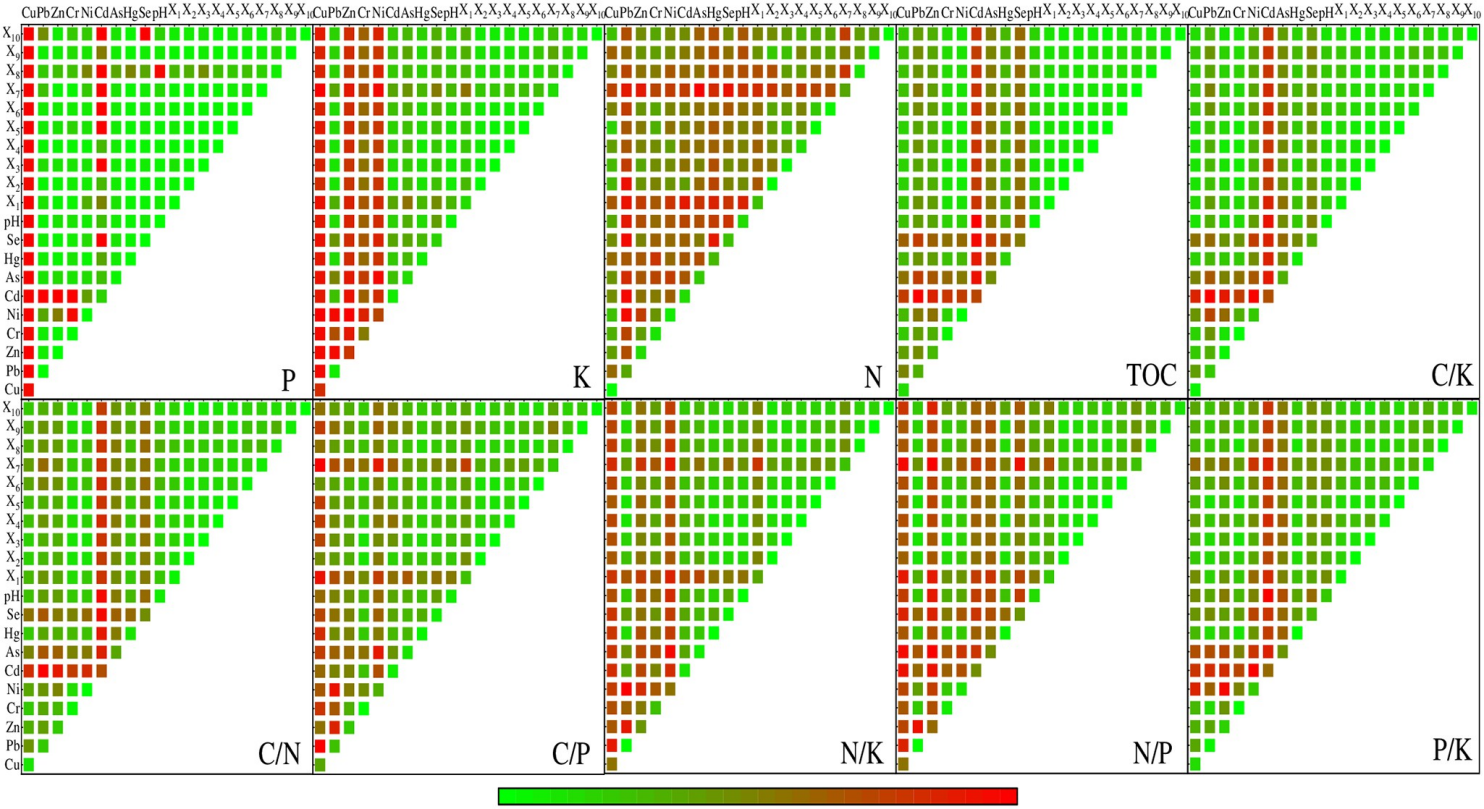
Interaction of different influence factors on oil nutrients and ecological chemometrics.

It can be seen from Fig 5 that the interaction of any two factors can explain the spatial heterogeneity of TOC, N, P, K, C/N, C/P, C/K, N/P, N/K and P/K to a greater extent than that of a single factor, most of which are nonlinear enhancement effects, and a few are dual factor enhancement effects. There is no weakening or independent action type. As far as P is concerned, the explanatory power of interaction between Cu with other 19 influencing factors is more than 0.8, the explanatory power of interaction between Cd with other 19 influencing factors is 0.84–0.14, and the explanatory power of interaction between X_8_ with other influencing factors is 0.82–0.15. The explanatory power of interaction is greatly increased, which is 2.5–13.7 times of its single factor explanatory power q value (0.060). For K, the explanatory power of interaction between Cu, Ni and Zn with other influencing factors is basically above 0.5, and the explanatory power of interaction between distance factors X_7_ and X_8_ with other influencing factors is significantly enhanced. For N, Pb, X_1_ and X_7_ have the strongest interaction with other17 influencing factors. For TOC, Cd, As and Se have the strongest interaction with other 17 influencing factors, and the explanatory power is more than 24%. For C/K, C/N and P/K, the interaction between Cd with other 19 influencing factors is the strongest. For C/P, Cu and Pb have the strongest interaction with other 18 influencing factors, and X_1_ and X_7_ have significantly enhanced the explanatory power of interaction with other 18 influencing factors, the explanatory power of interaction of X_1_ is 1.9–3.8 times that of its single factor, and the explanatory power of interaction of X_7_ is 1.3–4.7 times that of its single factor. For N/K, Cu, Zn and Ni have the strongest interaction with other 17 influencing factors, and X_1_ and X_7_ have significantly enhanced the explanatory power of interaction with other 18 influencing factors; For N/P, Cu, Zn, Cd, As and Se have the strongest interaction with other 15 influencing factors. In general, the interaction between soil properties with other 10 influencing factors has an important impact on the spatial differentiation of soil nutrients and their ecological chemometrics in the study area, and X_1_, X_7_ and X_8_ are also important factors affecting their spatial distribution.

#### Risk detector

**Fig 6:**
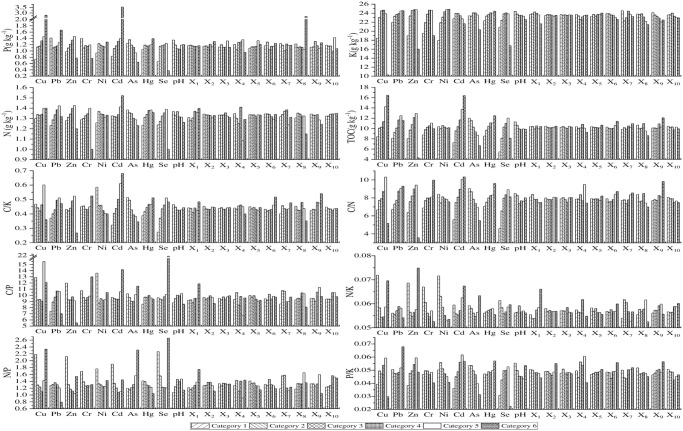
Risk detection of soil nutrients and ecological chemometrics.

The risk detector was used to detect whether there were significant differences in soil nutrients and their eco-chemometrics in each sub-region of 20 factors, as well as the high and low value areas of soil nutrients and their eco-stoichiometry in each factor sub-region. Fig 6 clearly shows the high and low value regions of TOC, N, P, K, C/N, C/P, C/K, N/P, N/K and P/K in each sub-region of 20 influence factors. For example, the high-value and low-value regions of P in the sub-region of influence factor Cd appear in the sixth and first type Cd respectively, while P in the sixth and fifth categories in the high and low value regions of X_8_ sub-regions, respectively.

As for P, its significant difference is strongest in As sub region, followed by significant difference in most sub regions of Cd, pH and Hg, while there is no significant difference in Cr, X_1_, X_3_ and X_9_ sub regions. For K, the difference is most significant in Ni sub region, followed by X_7_, Pb, Cr, As, Hg, pH and X_9_ sub region, while the difference is not significant in X_2_, X_3_ and X_4_ sub region. For N, there is no significant difference in the sub regions of each influencing factor, only in the partial molecular regions of Pb, Cd, As, Se and X_7_. For TOC, it has the most significant difference in Cd sub regions, followed by As, Cu, Pb, Zn, Hg, Se sub regions, while there is no significant difference in Ni, X_1_, X_2_, X_3_, X_4_, X_5_ and X_10_ sub regions. For C/K, it has the most significant difference in the sub regions of Cd, followed by Pb, Ni, As, Se, X_6_ and X_7_, while there is no significant difference in the sub regions of Cr, X_2_, X_3_, X_4_, X_5_ and X_10_. For C/N, its difference is most significant in the sub regions of As and Cd, followed by Cu, Hg and Se, while there is no significant difference in the sub regions of X_2_, X_3_, X_4_, X_5_ and X_10_. For C/P, its difference is most significant in the sub regions of Pb and X_7_, while there is no significant difference in the sub regions of Cr, X_2_ and X_3_. For N/K, its difference is most significant in Ni, X_1_ and X_7_ sub regions, while there is no significant difference in Hg, X_2_, X_3_, X_5_, X_6_, X_8_, X_9_ and X_10_ sub regions. For N/P, the sub regions of As, pH, X_1_ and X_7_ have the most significant differences, and only the sub regions of X_3_ have no significant differences. For P/K, the difference is strongest in the sub regions of Cd, followed by Cd, pH, Ni and X_6_, while there is no significant difference in the sub regions of X_3_ and X_5_.

#### Ecological detector

The ecological detector focuses on comparing whether there is a significant difference between one influence factor and another on the spatial distribution of soil nutrients and their ecological chemometrics [31, 32].

The ecological detection results of soil nutrients and their ecological stoichiometry in the study area showed that there were significant differences in the effects of Cu, Pb, Zn, Cr, Ni, Cd, As, Hg, Se and pH with X1 for K, and that Pb, Zn, Cr, Ni, Cd, Hg, Se and pH have significant differences with X_1_ on N/K; the Pb, Zn, Cr, Ni, Cd, As, Hg and Se with X_1_ and X_3_ with X_7_ have influences on N/P significant difference, but not significant difference among other factors.

## Discussion

The order of impact of each influencing factor on TOC, N, P, K, C/N, C/P, C/K, N/P, N/K and P/K is different, revealing different soil nutrients and their ecological stoichiometry, the heterogeneity of the mechanism of chemical change is caused by the joint action of various influencing factors. In general, the main factors affecting the spatial distribution of soil and its ecological stoichiometry are soil properties. Large-scale planting of fruits and vegetables and horticultural nurseries in the study area, agricultural activities such as the use of nutrient soil, fertilization, irrigation, and pesticide application directly make the soil C, N, P, and K change, which in turn leads to changes in its ecological stoichiometry [33, 34]. Correlation analysis also showed that soil nutrients and ecological stoichiometry had extremely significant correlations with soil heavy metals [35]. It is mainly due to the good inhibitory effect of nutrients on the toxicity of some heavy metal elements. in addition, many chemical fertilizers or pesticides contain some heavy metal elements as well as a large amount of C, N, P, K components [36]. As one of the important indicators of soil physical and chemical properties, pH has weak explanatory power to the spatial heterogeneity of soil nutrients and eco-stoichiometry in the study area, which may be due to the weak soil pH and small variability in the study area.

Topography is one of the important factors affecting soil development, which affects soil nutrients by regulating the spatial redistribution of soil moisture and solar radiation [37]. The elevation of the study area has an obvious influence on the spatial distribution of K, N, C/P, N/K and N/P, but the effects of slope and aspect on soil nutrients and their ecological chemometrics are not obvious, which may be related to the small area of the study area and the small variation range of slope and aspect. Land use types have no significant effect on the spatial distribution of soil C, N, P and K and their ecological stoichiometry, which is significantly different from other people’s studies [38, 39], which may be due to frequent changes in land use types in the study area (such as rapid changes in agricultural land and horticultural nursery land) and the lack of obvious boundaries in the study area.

Human activities have changed the distribution characteristics of soil nutrients and their eco-stoichiometric space in the natural state, forming new spatial characteristics. The enrichment of water source P, N, K is caused by industrial wastewater discharge and domestic sewage discharge, and then farmland soil enrichment is caused by agricultural irrigation [40–42]. Relevant studies have pointed out that residential areas are the areas with the most frequent human activities. Residents will produce a large amount of domestic waste containing C, N, P and K in their daily life, which will cause changes in the soil around residential areas, at the same time, frequent human activities in cities and towns will make some C, N, P and K are enriched into the soil through diffusion methods such as atmospheric deposition, which leads to changes in soil nutrients and their ecological stoichiometry [43–45]. In addition, factories and enterprises such as building materials, plastics and printing are distributed in the study area, the C, N, P and K carried by the waste generated by industrial activities are enriched into the soil through atmospheric deposition, rainwater erosion and infiltration. It is found that the distance from the railway has the strongest explanation for soil N, but there is a difference between the two results by comparing the Pearson correlation analysis, that is, there is no significant correlation between the distance from the railway and soil N, this is because the geographic detector analyzes the correlation between soil nutrients and influencing factors, including linear and nonlinear relationships, while the Pearson correlation coefficient is not significant, indicating that there is no significant linear relationship between soil nutrients and influencing factors, but that doesn’t mean there’s no nonlinear relationship [24].

The factor detector can achieve good results in explaining the spatial variation of soil nutrients (Fig 2), but from the specific explanatory power value, it is obvious that these factors cannot fully explain the variation of nutrients. Therefore, many factors of soil formation and change should be considered comprehensively, and representative and stronger influencing factors should be extracted, such as soil parent material [38], soil type [38], vegetation type [39], soil bulk density [46], irrigation method [47], crop rotation method [48] and climate and environmental shadows [38], to a greater extent explain the spatial variability of soil nutrients and their ecological stoichiometry.

## Conclusions

The spatial differences of farmland soil nutrients and their ecological chemometrics in Tangchang Town, National Agricultural Park, were studied by using geographical detectors. The main conclusions are as follows:

1. The spatial distribution of soil nutrients and their ecological chemometrics in the study area was significantly different. The high value of P was distributed in the northern and southern marginal areas, and the high value of K was distributed in the areas except for a few villages in the west and northeast; the high value area of N is mainly concentrated in the northwest of the study area, and the high value area of TOC is concentrated in the middle and northeast. The high value areas of C/P, N/K and N/P are mainly concentrated in the west of the study area; The high value areas of C/N and P/K are mainly distributed in northeast; the high value areas of C/K are mainly distributed in the northeast, northwest and west.
2. Correlation analysis shows that most of soil nutrients and their ecological chemometrics have significant correlation with each other, and the correlation with soil properties is the strongest, followed by the correlation with distance factors and topographic factors, and the correlation with soil forming factors is the weakest.
3. The factor detection results show that soil pH, Cd, Hg, As, Cu, Pb, Cr, Zn, Ni and Se, as well as the distance from the railway and the residential area have a particularly significant explanatory power on the spatial heterogeneity of soil nutrients and ecological chemometrics. The interactive exploration found that the explanatory power of interaction showed an enhanced effect. The interaction between soil properties and other factors was the most important factor affecting the spatial differentiation of soil nutrients and their ecological chemometrics. Elevation, distance from railway and residential area were also important factors. The risk detection shows that the differences of soil nutrients and their ecological chemometrics are most significant in the sub regions of influencing factors pH, Cd, Hg, As, Cu, Pb, Cr, Zn, Ni and Se. In general, the spatial distribution of soil nutrients and their ecological chemometrics in the study area is the result of the joint action of many factors, and different influencing factors have different effects on different soil nutrients and their ecological chemometrics.

## Supporting information

S1 Data Figs 4–6 supporting data. (XLSX)
